# Grain yield and quality performances of different late-season rice cultivars in response to experimental warming in subtropical China

**DOI:** 10.3389/fpls.2023.1136564

**Published:** 2023-05-15

**Authors:** Taotao Yang, Xueming Tan, Shan Huang, Xiaohua Pan, Yongjun Zeng, Jun Zhang, Shanmei Cheng, Yanhua Zeng

**Affiliations:** ^1^ Ministry of Education and Jiangxi Key Laboratory of Crop Physiology, Ecology and Genetic Breeding, Jiangxi Agricultural University, Nanchang, China; ^2^ Rice Research Institute/Guangdong Key Laboratory of New Technology in Rice Breeding/Guangdong Rice Engineering Laboratory, Guangdong Academy of Agricultural Sciences, Guangzhou, China; ^3^ Institute of Crop Sciences, Chinese Academy of Agricultural Sciences, Beijing, China

**Keywords:** climate warming, late-season rice, growth duration, head rice yield, grain quality

## Abstract

**Introduction:**

Climate warming has pronounced effects on rice production in China. However, late-seasons rice cultivars are diverse in double rice cropping systems, and the actual responses in grain yield and quality of different late-season rice cultivars to climate warming are still unclear.

**Methods:**

A two-year field warming experiment was conducted by using free-air temperature increase facilities with three widely-planted late-season rice cultivars, including Taiyou398 (TY, short growth duration *indica* hybrid rice), Jiuxiangnian (JXN, long growth duration *indica* inbred rice), and Yongyou1538 (YY, long growth duration *indica-japonica* hybrid rice) in a double rice cropping system in subtropical China.

**Results:**

Warming (1.9–2.0°C) had no significant effects on the grain yields of TY and JXN, but significantly decreased that of YY by 4.8% relative to ambient treatment due to a reduction of spikelet number. Compared to ambient treatment, the head rice yields of TY and YY did not change while that of JXN increased by 6.3% under warming conditions. Warming significantly increased the head rice rates of JXN and YY by 6.6% and 7.8%, and the chalky grain rates of TY, JXN, and YY by 79.1%, 21.6%, and 7.6%, respectively. Under warming conditions, the amylose content of JXN and YY decreased significantly by 7.5% and 8.8%, and the setback of three cultivars decreased significantly by an average of 41.5%.

**Conclusion:**

Warming could improve the milling and eating qualities of long growth duration late-season rice (JXN and YY) and increase or maintain their head rice yield, even though decreased the grain yield of *indica-japonica* hybrid rice (YY). These results will provide a better understanding for the selection of suitable late-season rice cultivars under future climate warming conditions.

## 1 Introduction

Rice (*Oryza sativa* L.), as a primary source of caloric intake, is the most important staple food for more than half of the world’s population. China is the world’s largest rice production and consumption country with a rice cultivation area of approximately 30 million hectares and a grain yield of approximately 212 million tons in 2020 (NBS., 2021). Compared to 1850–1900, the global surface temperature averaged over 2041–2060 is very likely to be higher by 1.6°C to 2.5°C in the intermediate greenhouse gas emissions scenario (IPCC, 2021). Abnormal surface temperatures will adversely cause stresses on rice growth and ultimately affect grain yields in China (Chen et al., 2020; Liu et al., 2020). Therefore, it is crucial to evaluate the actual responses in rice production to climate warming in China.

Similar to solar radiation, free-air temperature increase (FATI) facilities directly shed infrared on vegetation and are widely used in terrestrial ecosystem warming experiments (Bai et al., 2013). In recent years, numerous FATI experiments have been conducted to reveal the actual responses of grain yields to climate warming in China (Cai et al., 2016; Wang et al., 2016; Wang et al., 2018b). Chen et al. (2020) found that warming increased the grain yields in single rice cropping systems in the northeast of China, whereas decreased those in middle rice cropping systems in central and eastern China, and had contrasting effects for early- and late-season rice yields in double rice cropping systems in South China. The grain yields in response to warming vary strongly in the three major rice cropping systems, which may be due to the different air temperature and precipitation patterns across China (Chen et al., 2020).

Rice quality refers to milling, appearance, nutritional, and eating qualities, and is an important criterion for acceptance of rice cultivar by farmers and consumers. Warming affects various rice physiological processes regarding grain quality such as assimilate accumulation and transport, grain filling, and starch synthesis (Wang et al., 2015; Chen et al., 2017; Tang et al., 2019). Previous studies have indicated that warming by using FATI facilities increased chalky grain rate and chalkiness but decreased milled and head rice rate, suggesting that warming worsened rice milling and appearance qualities (Dou et al., 2017; Tang et al., 2019; Yang et al., 2022b). The deterioration of grain milling quality under warming conditions leads to a decrease in head rice yield, which further reduces the edible rice yield and the economic value of rice from field to market (Lyman et al., 2013). Meanwhile, warming by using FATI facilities reduced the amylose content in rice grains, altered the pasting property of rice flour, and resulted in changes in rice cooking and eating quality (Dou et al., 2017; Dou et al., 2018; Tang et al., 2019). However, most of these previous studies regarding the effect of warming on rice quality were conducted in single rice cropping systems (Hu et al., 2021; Hu et al., 2022). To the best of our knowledge, the impact of climate warming on grain quality has barely been reported in double rice cropping systems.

As one of the most important rice cropping systems in China, the planting area and grain yield of double-cropping rice accounted for 33.0% and 27.5% in 2020, respectively (NBS, 2021). In double rice cropping systems, the growth duration of late-season *indica* rice cultivars range approximately from 110 to135 days. Rice cultivars with different growth durations are expected to get different light and temperature resources, especially during grain-filling stage. Warming changed the rice phenophase with obviously different meteorological features, resulting in the changes in grain yields (Chen et al., 2017; Wang et al., 2018b). Recently, “*indica* rice to *japonica* rice” engineering has been implemented in South China to ensure food security. “*Indica* rice to *japonica* rice” refers to *indica-japonica* hybrid rice, pure *japonica* hybrid rice, and *japonica* inbred rice cultivars instead of *indica* rice cultivars planted as late-season rice. Compared to *indica* hybrid rice and *japonica* inbred rice, the grain yields of *indica*-*japonica* hybrid rice are significantly higher because of high canopy light capture capability and full use of solar radiation in late rice seasons (Yin et al., 2021). Due to the diversity of late-season rice cultivars in double rice cropping systems, it is necessary to quantify the impacts of climate warming on the grain yields and qualities of different late-season rice cultivars, which is crucial for ensuring staple rice supply in China.

We hypothesized that the effects of warming on grain yields and qualities of different late-season rice cultivars are different. In this study, a field warming experiment was conducted with three different rice cultivars by using FATI facilities in late rice seasons in 2018 and 2019. Our objectives were to evaluate the actual responses of grain yields and qualities to experimental warming and to quantify the different responses among the three late-season rice cultivars.

## 2 Materials and methods

### 2.1 Site descriptions

The field experiment was conducted in 2018 and 2019 at the experimental base (115°09’E, 28°31’N) in Shanggao County, Jiangxi Province, China. Double rice cropping systems consist of early-season rice and late-season rice followed by winter fallow period. Late-season rice is growing from June sowing to November harvesting. The daily average temperature, daily precipitation, and sunshine duration during the late rice seasons in 2018 and 2019 are presented in **Figure S1**. The topsoil (0–15 cm) properties before the experiment in 2018 were: pH of 5.5, organic carbon concentration of 20.5 g kg^-1^, total nitrogen concentration of 2.0 g kg^-1^, alkaline hydrolyzable nitrogen concentration of 190.1 mg kg^-1^, available phosphorus concentration of 20.0 mg kg^-1^, and available potassium concentration of 65.1 mg kg^-1^.

### 2.2 Experimental design

**Figure 1:**
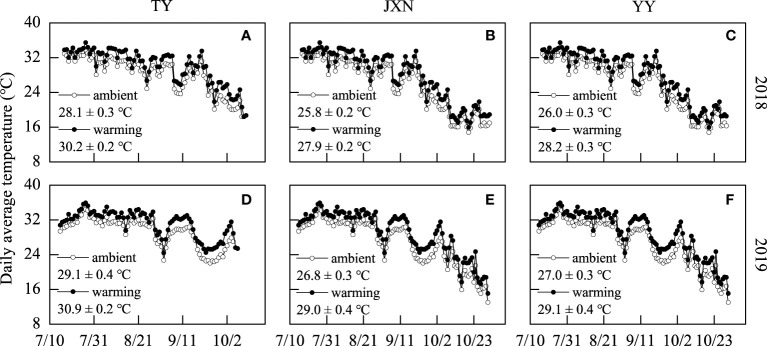
Daily average temperature of rice canopy under two temperature treatments during the rice growth period. TY, JXN and YY indicate Taiyou398, Jiuxiangnian and Yongyou1538, respectively. Upper panel was TY **(A)**, JXN **(B)** and YY **(C)** in 2018, and lower panel was TY **(D)**, JXN **(E)** and YY **(F)** in 2019. The temperature values displayed in the figure stand for the average temperatures of the whole growth period. Mean ± standard deviation (n=3).

There were two temperature treatments: (1) ambient temperature treatment (ambient) and (2) experimental warming treatment (warming), with three replicates for each treatment with random arrangement. Each plot was 5 m wide and 10 m long. Details of the FATI system were reported in our previous study (Yang et al., 2020). Briefly, one infrared heater (1500 W, 180 cm in length, 20 cm in width) was suspended 75 cm above rice canopy (in the middle of last leaf) in each warming plot for a cultivar. A ‘dummy’ heater with the same size was suspended to imitate the shading effects of the heater. Each infrared heater formed a 1.5 m × 1.8 m sampling area with uniform and reliable warming effects (**Figure S2**). Warming treatments carried out from transplanting to maturity. The rice canopy temperature of each cultivar was monitored by a digital temperature monitor (ZDR–41, Hangzhou Zeda Electronic Instrument, Hangzhou, China) in 1 h intervals. The daily average temperature of the rice canopy under two temperature treatments are shown in **Figure 1**. Compared to ambient treatments, warming increased the daily average temperature of TY, JXN, and YY by 1.9°C, 2.1°C, and 2.1°C over the two years, respectively.

### 2.3 Crop management

The tested late-season rice cultivars were Taiyou398 (TY), Jiuxiangnian (JXN), and Yongyou1538 (YY), which are popular rice cultivars in Jiangxi Province, China. Taiyou398 is an *indica* hybrid rice cultivar with a short growth duration of approximately 110 d; JXN is an *indica* inbred rice cultivar with a long growth duration of approximately 135 d; YY is an *indica-japonica* hybrid rice cultivar with a long growth duration of approximately 132 d. The sowing, transplanting, heading, and maturity dates of the three rice cultivars are showed in **Table S1**. Rice seedlings were manually transplanted at a hill space of 25 cm × 13 cm with three seedlings per hill. Urea (46.0% N concentration), calcium magnesium phosphate (12.0% P_2_O_5_ concentration), and potassium chloride (60.0% K_2_O concentration) were used as nitrogen, phosphorus, and potassium fertilizers, respectively. The application rates of the nitrogen, phosphorus, and potassium fertilizers were 210.0, 46.5, and 156.2 kg ha^-1^ in late rice seasons, respectively. Nitrogen fertilizer was applied in three splits, 40% was applied as basal fertilizer (1 day before transplanting), 20% was top-dressed at early tillering (7 days after transplanting), and 40% was top-dressed at panicle initiation. All of the phosphorus fertilizer and 70% potassium fertilizer were applied as basal fertilizers with the remaining potassium fertilizer applied at panicle initiation. The field was kept flooded from transplanting to mid-season drainage, and was then intermittently irrigated until maturity. Other field management measures, including diseases, weeds, and insects were the same as double cropping rice production.

### 2.4 Measurements

#### 2.4.1 Grain yield and yield components

At physiological maturity, the panicle number were counted with 40 hills rice plants in each plot. Based on the average panicle number, five hills of rice plants were taken from each plot to measure the spikelet number, filled grain percentage, and grain weight. Finally, 40 hills rice plants that were located directly below the infrared heater in the reliable warming area were manually harvested in each plot to determine the grain yield. The rice samples were stored at the room temperature for three months before the determination of grain qualities.

#### 2.4.2 Head rice rate, chalky grain rate, and head rice yield

The determination of head rice rate and chalky grain rate were conducted based on the national standard for rice quality evaluation (GB/T 17891–2017), People’s Republic of China (NBQTC, 2017). Briefly, 120 g of rice grain samples were dehulled with a rice huller, and then brown rice was milled with a rice polisher. The grain quality inspector equipped with image analysis software (SC-E, Wanshen Technology Company, Hangzhou, China) was used to measure the head rice rate and chalky grain rate (i.e., the percentage of chalky rice grains in the total sample) of milled rice. The head rice yield was calculated with the following equation:


$$Head\;rice\;yield\;\left(t\;hm^{-2}\right)\;=\;Grain\;yield\;\left(t\;hm^{-2}\right)\times Head\;rice\;rate\;\left(\%\right)$$

#### 2.4.3 Amylose content and rapid viscosity analyzer parameters

After the measurement of chalky grain rate, milled rice samples were ground and sieved (60-mesh) for subsequent analysis. The amylose was determined according to the national standard for rice quality evaluation (GB/T 17891–2017), People’s Republic of China (NBQTC, 2017). Rice flour RVA parameters were determined by a rapid viscosity analyzer (RVA–Super 4, New Scientific, Warriewood, Australia) according to the protocol from the American Association for Cereal Chemistry (AACC, 1999). Briefly, 3 g of rice flour was thoroughly mixed with 25 ml of ultrapure water in an aluminum cylinder. The sample was heated at 50°C for 1 min, then heated from 50 °C to 95°C and cooled to 50°C at a rate of 12°C min^-1^. The peak viscosity, trough viscosity, final viscosity, breakdown viscosity (peak viscosity–trough viscosity), and setback viscosity (final viscosity–peak viscosity) were recorded.

### 2.5 Statistical analysis

All statistical analyses were carried out using IBM SPSS ver. 24.0. We analyzed the grain yield, yield components, and grain quality by using three-way analyses of variance (ANOVA). The treatments for each cultivar in the same year were compared using Student’s t-test and the differences were determined to be statistically significant when 0.01< *P* ≤ 0.05 and *P* ≤ 0.01.

## 3 Results

### 3.1 Grain yield, head rice yield, and yield components

**Figure 2:**
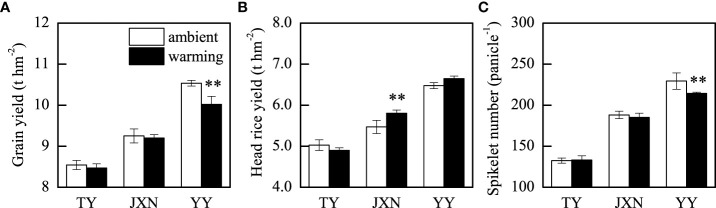
Grain yield **(A)**, head rice yield **(B)**, and spikelet number **(C)** as affected by temperature and cultivar. These variables showed significant temperature × cultivar interactions, so data were presented for each cultivar. TY, JXN, and YY indicate Taiyou398, Jiuxiangnian, and Yongyou1538, respectively. Error bars represent the standard deviation of the mean. Asterisks indicate significant differences between temperature treatments within the same cultivar at 0.001< *P* ≤ 0.01 (**) or 0.01< *P* ≤ 0.05 (*).

Averaged across cultivars and years, warming significantly reduced grain yield by 2.2% and the spikelet number by 3.1% but had no effect on head rice yield, panicle number, filled grain percentage, and grain weight (**Table 1**). Grain yield, head rice yield, and yield components varied among the three cultivars. Temperature and cultivar had interactive effects on grain yield, head rice yield, and spikelet number (**Table 1**). Warming had no effect on the grain yields and spikelet number of TY and JXN but significantly reduced those of YY by 4.9% and 6.5%, respectively (**Figures 2A, C**). The head rice yield of JXN was significantly increased by 5.3%, while that of TY and YY were not changed under warming conditions (**Figure 2B**). In addition, grain yield, head rice yield and spikelet number varied with experimental years. Grain yield and spikelet number were higher and head rice yield was lower in 2018 than those in 2019 (**Table 1**).

**Table:** TABLE 1 Grain yield, head rice yield, and yield components as affected by temperature, cultivar, and study year.

	Panicle number (m^-2^)	Spikelet number per panicle	Filled grain percentage (%)	Grain weight (mg)	Grain yield (t hm^-2^)	Head rice yield (t hm^-2^)
Temperature (T)^a^						
ambient	296.9a	183.3a	90.7a	25.08a	9.44a	5.66a
warming	304.5a	177.6b	89.8a	24.94a	9.23b	5.78a
Cultivar ^©b^						
TY	353.9a	132.9c	89.8b	26.28a	8.51c	4.96c
JXN	310.9b	186.7b	87.8b	22.37b	9.23b	5.64b
YY	237.4c	221.9a	93.2a	26.39a	10.28a	6.56a
Year (Y)^c^						
2018	303.1a	183.9a	90.7a	25.01a	9.54a	5.58b
2019	298.4a	177.1b	89.9a	25.02a	9.14b	5.86a
*F* values						
T×C	1.32	4.47^*^	1.23	0.70	4.12^*^	4.40^*^
T×Y	1.46	0.17	0.30	0.03	1.75	4.05
C×Y	6.30^*^	3.01	2.29	0.54	8.62^**^	0.21
T×C×Y	0.81	1.20	0.45	0.62	4.65^*^	1.30

TY, JXN, and YY indicate Taiyou398, Jiuxiangnian, and Yongyou1538, respectively. Different lowercase letters in the same column indicate significant differences in the main effect of temperature, cultivar, or year. Significant interactive effects are indicated by ^*^ (0.01< P ≤ 0.05) or ^**^ (0.001< P ≤ 0.01).

a Values were averaged across cultivars and years.

b Values were averaged across temperature treatments and years.

c Values were averaged across temperature treatments and cultivars.

### 3.2 Head rice rate and chalky grain rate

**Figure 3:**
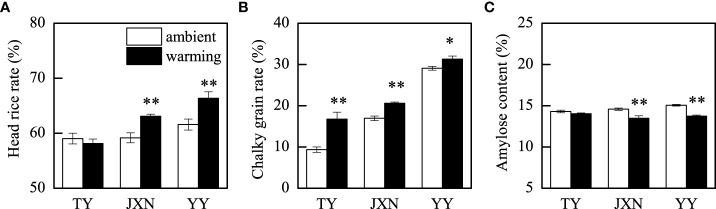
Head rice rate **(A)**, chalky grain rate **(B)**, and amylose content **(C)** as affected by temperature and cultivar. These variables showed significant temperature × cultivar interactions, so data were presented for each cultivar. TY, JXN, and YY indicate Taiyou398, Jiuxiangnian, and Yongyou1538, respectively. Error bars represent the standard deviation of the mean. Asterisks indicate significant differences between temperature treatments within the same cultivar at 0.001< *P* ≤ 0.01 (**) or 0.01< *P* ≤ 0.05 (*).

Compared to ambient treatment, warming significantly increased head rice rate and the chalky grain rate by 4.3% and 23.8%, respectively (**Table 2**). Head rice rate and chalky grain rate varied among cultivars and experimental years, and significant interactions between temperature and cultivar were observed to affect head rice rate and chalky grain rate (**Table 2**). Warming significantly increased the head rice rate by 6.6% of JXN and by 7.8% of YY, whereas no significant effect was observed on TY (**Figure 3A**). The chalky grain rates increased significantly by 79.1%, 21.6%, and 7.6% of TY, JXN, and YY under warming conditions, respectively (**Figure 3B**). Furthermore, head rice rate was lower and chalky grain rate was higher in 2018 than in 2019 (**Table 2**).

**Table:** TABLE 2 Head rice rate, chalky grain rate, amylose content, and RVA parameters as affected by temperature, cultivar, and study year.

	Head rice rate (%)	Chalky grain rate (%)	Amylose content (%)	RVA parameters (cP)		
				Peak viscosity	Breakdown	Setback
Temperature (T)^a^						
ambient	59.9b	18.5b	14.6a	3237a	1415a	-195a
warming	62.5a	22.9a	13.7b	3282a	1433a	-276b
Cultivar (C)^b^						
TY	58.6c	13.1c	14.2ab	3369a	1548a	-372b
JXN	61.1b	18.8b	14.0b	3192b	1494a	-317b
YY	64.0a	30.2a	14.4a	3218b	1230b	-18a
Year (Y)^c^						
2018	58.4b	28.3a	14.5a	3216b	1504a	-274b
2019	64.0a	13.0b	13.8b	3303a	1343b	-197a
*F* values						
T×C	12.00^**^	14.18^**^	6.79^**^	0.66	1.88	0.93
T×Y	14.32^**^	7.54^*^	9.74^**^	0.00	2.64	3.72
C×Y	13.32^**^	185.38^**^	0.22	25.29^**^	24.53^**^	35.42^**^
T×C×Y	6.92^**^	4.88^*^	1.37	0.56	1.12	0.32

TY, JXN, and YY indicate Taiyou398, Jiuxiangnian, and Yongyou1538, respectively. Different lowercase letters in the same column indicate significant differences in the main effect of temperature, cultivar, or year. Significant interactive effects are indicated by ^*^ (0.01< P ≤ 0.05) or ^**^ (0.001< P ≤ 0.01).

a Values were averaged across cultivars and years.

b Values were averaged across temperature treatments and years.

c Values were averaged across temperature treatments and cultivars.

### 3.3 Amylose content and RVA parameters

Amylose content and setback were decreased by 6.2% and 41.5%, respectively, under warming conditions relative to the ambient treatment, whereas peak viscosity and breakdown were not significantly affected by warming (**Table 2**). Amylose content and RVA parameters varied with cultivars and experimental years. Significant interactive effect of temperature and cultivar on amylose content was observed, and on RVA parameters was not found. As shown in **Figure 3C**, warming had no significant effect on amylose content of TY but significantly decreased that by 7.5% and 8.8% of JXN and YY, respectively. Compared to those in 2019, amylose content and breakdown were higher and peak viscosity and setback were lower in 2018 (**Table 2**).

## 4 Discussion

### 4.1 The grain yields and head rice yields of different late-season rice cultivars in response to warming

The grain yields in response to warming varied strongly among the late-season rice cultivars (**Table 1**). Differently, previous studies found that warming by using FATI facilities significantly reduced the grain yields for both *indica* rice (Rehmani et al., 2014) and *japonica* rice (Cai et al., 2016; Wang et al., 2016; Wang et al., 2018a; Wang et al., 2018b) in middle rice cropping systems. The negative effects of warming on grain yields were due to the decrease in panicle number (Wang et al., 2018b), spikelet number, filled grain percentage, and grain weight (Rehmani et al., 2014; Cai et al., 2016). In this study, the grain yields of *indica* rice (TY and JXN) did not change under warming conditions, which was attributed to the minor changes in yield components, while warming reduced the spikelet number of *indica*-*japonica* rice (YY), resulting in a decrease in its grain yield (**Figure 2A**).

The impact of warming on grain yield formation depends on ambient air temperature (Chen et al., 2017; Chen et al., 2020). The optimal temperature for rice tillering is approximately 28.4°C, ranging from 16.4°C to 35.3°C; for rice anthesis is approximately 26.3°C, ranging from 16.2°C to 37.0°C; and for grain-filling is approximately 24.2°C, ranging from 20.7°C to 31.3°C (Sánchez et al., 2014). In this study, the canopy average temperature of ambient treatments was 31.6°C to 32.1°C from transplanting to panicle initiation, 26.8°C to 29.4°C from initial heading to full heading, and 20.5°C to 26.4°C from full heading to maturity (**Table S2**). Thus, experimental warming (1.9–2.0°C) did not exceed the upper temperatures range limit for the three late-season rice cultivars and had little effects on their panicle number, filled grain percentage, and grain weight.

The spikelet number in response to warming were different between *indica* rice (TY and JXN) and *indica*-*japonica* rice (YY) in this study (**Table 1**). Compared to *indica* rice cultivars, the growth of *indica-japonica* rice cultivars is obviously sensitive to temperature changes of in late rice seasons (Yin et al., 2021). In addition, we found that warming was not conducive to the dry matter accumulation at the heading stage of *indica-japonica* rice, especially the dry matter accumulation of the panicle (**Figure S3**). Therefore, compared to *indica* rice cultivars (TY and JXN), *indica-japonica* hybrid rice (YY) might have poor heat resistance in this study. Warming might adversely affect the panicle differentiation or increase the ratio of spikelet degeneration and led to the decrease of spikelet number of YY (**Figure 2C**). In addition, the canopy average temperature from panicle initiation to initial heading was 30.5°C in 2018 and 31.1°C in 2019 (**Table S2**). The higher temperature might be may be detrimental to panicle differentiation (Sánchez et al., 2014), resulting in lower spikelet number and grain yield in 2019. Consequently, climate warming will make temperatures unfavorable for *indica-japonica* rice (YY) production in the future. It is important to study the direct effects of warming on spikelet number and find countermeasures to prevent the yield reduction of late-season *indica-japonica* rice under future climate warming conditions.

High temperature on rice production focus on the grain yield and fail to account for the detrimental impact of high temperature on milling quality, which ultimately determine the head (edible) rice yield and market value. Lyman et al. (2013) found that warming (1.0°C) during growing season significantly reduced grain yield and further reduced head rice yield due to the increased percentages of chalkiness and broken kernels. Different with grain yields, the head rice yields of TY and YY were not changed under warming conditions, while the head rice yield of JXN was increased (**Figure 2B**). As discussed below, under warming conditions, the increase in the head rice rates of JXN and YY resulted in an increase in head rice yield of JXN and compensated for the reduction in head rice yield of YY (**Figure 3A**). In total, our results highlighted that the importance of planting long growth duration late-season rice cultivars (e.g., JXN and YY) in double rice cropping systems to maintain or increase the head rice yield and bolster food security under future climate warming conditions.

### 4.2 The grain qualities of different late-season rice cultivars in response to warming

Generally, no matter what kinds of temperature increase facilities are used, experimental warming or high temperature during grain-filling stage always increases chalky grain rate and chalkiness and worsens rice appearance quality (Lanning et al., 2011; Xiong et al., 2017; Wang et al., 2021). In this study, this phenomenon was also confirmed by using three late-season rice cultivars under field warming conditions (**Table 2**). There were several reasons for the cause of warming-induced grain chalkiness in this study. First, in the initial stage of grain-filling, high temperature accelerates the grain-filling rate, resulting in the loose packing of amyloplast (Dou et al., 2017). Second, high temperature-induced α-amylase is involved in the degradation of endosperm starch granules, forming hydrolysis traces, such as small pits, leading to an increase in chalkiness (Hakata et al., 2012). Third, warming tends to increase protein and amino acid contents and disturbs the accumulation balance of storage protein, amino acid, and starch, which coordinately controls the formation of grain chalkiness (Dou et al., 2018; Tang et al., 2019; Wang et al., 2021). Compared to short growth duration rice cultivar (TY), warming had less effects on the chalky grain rates of long growth duration rice cultivars (JXN and YY), might be due to their lower ripening temperature from full heading to maturity (**Table S2**) and longer grain-filling stage (53–58 d, **Table S1**), which were beneficial to alleviate the adverse effects of warming on grain-filling. Furthermore, the higher chalky grain rate in 2018 might be attributed to the increased daily precipitation and decreased sunshine duration from heading to maturity (**Figure S1**).

Rice is consumed primarily as intact kernels, and the head rice rate primarily determines market prices and producer revenues. Our results showed that head rice rate was significantly increased by warming but varied among the three late-season rice cultivars (**Table 2**; **Figure 3A**), which were different with previous reports (Rehmani et al., 2014; Jing et al., 2016; Dou et al., 2018; Tang et al., 2019). In general, high ripening temperature decreases head rice rate due to the increase in chalky kernel percentage (Wang et al., 2015; Siddik et al., 2019). The different observations in this study might be caused by the type and location of chalk formation, which is crucial to the degree of kernel breakage during milling (Lyman et al., 2013). According to Lyman et al. (2013), high temperature corresponding to the formation of white back or basal white chalky kernels did not result in an equivalent reduction in head rice rate. The optimal temperature for grain-filling is approximately 24.2°C (Sánchez et al., 2014). In the present study, the ambient temperature in late grain-filling stages were very low (**Figure 1**). Therefore, warming (1.9–2.0°C) might be beneficial to the grain-filling and promote the maturity of inferior grains, especially for JXN and YY, reducing the broken kernel percentage during processing (Dou et al., 2017; Dou et al., 2018). In this study, we concluded that the chalk formed under warming conditions did not directly lead to the kernel breakage but would reduce the market value of head rice due to an increase in chalky grain rate. Consequently, planting long growth duration *indica* inbred rice cultivar (JXN) and *indica-japonica* hybrid rice cultivar (YY) in double rice cropping systems could improve the milling quality and reduce the deterioration of appearance quality under warming conditions.

It is generally acknowledged that the lower the amylose content, the better the taste of cooked rice (Chen et al., 2021). Our results indicated that warming decreased the amylose contents in head rice (**Table 2**), which were in agreement with previous studies (Chun et al., 2015; Dou et al., 2018; Tang et al., 2019). Amylose in rice endosperm is synthesized by granule-bound starch synthase (GBSS), encoded by the Waxy gene (Huang et al., 2021). High temperature-induced the decrease in amylose content is mainly attributed to the reduction of GBSS activity (Ahmed et al., 2015; Cao et al., 2015). Besides, we noted that the amylose content of TY in response to experimental warming was relatively smaller, which might due to a high heat resistance of short growth duration *indica* hybrid rice (**Figure 3C**).

The RVA parameters of rice flour are useful tools to assess the eating quality of cooked rice (Chen et al., 2021). Setback from RVA, also known as short-term retrogradation of rice flour, is positively correlated with the hardness of cooked rice (Li et al., 2020). In general, cooked rice with better taste quality always has higher values of peak viscosity and breakdown and lower setback value (Chen et al., 2021). Therefore, warming was expected to improve the rice eating quality, as it did not affect the peak viscosity and breakdown but significantly decreased the setback, especially for long growth duration rice cultivars (JXN and YY) (**Table 2**). Amylose content is an important factor in determining retrogradation characteristic because of the fast recrystallization rate of amylose molecules (Li et al., 2020). Thus, the lower setback values of rice flour under warming conditions might attribute to the decrease in amylose contents in the present study. Besides amylose content, other chemical compositions such as protein also negatively affect rice eating quality (Chen et al., 2021). Previous studies have reported that protein content in milled rice was enhanced under warming conditions (Dou et al., 2017; Dou et al., 2018; Yang et al., 2022b). Although the amylose content and RVA parameters indicated that warming improved the eating quality of late-season rice, the increase in protein content may reduce the eating quality. Thus, the actual effect of warming on the eating quality of late-season rice in the future should be evaluated by human tasting (Kim et al., 2017). Moreover, the fine structure of rice starch also affects the pasting property and eating quality (Tao et al., 2018; Chen et al., 2021), and its mechanism needs to be further studied under warming conditions.

In short, experimental warming has adverse effects on the grain yield, milling and appearance quality of late-season rice. Selecting suitable rice cultivars can compensate the adverse effects of warming on grain yield and quality of late-season rice. Other climate-smart strategies including innovative crop management and breeding should be developed to alleviate the negative impact of warming on rice production (Xu et al., 2021). For example, previous studies have shown that optimizing the rate and timing of mineral nitrogen fertilizer application, can also mitigate the adverse effects of warming on grain yield and quality (Tang et al., 2019; Yang et al., 2022a).

## 5 Conclusions

The grain yields, head rice yields, and grain qualities in response to warming varied among the three late-season rice cultivars. Warming decreased the grain yield of *indica-japonica* rice YY but had no significant effects on those of *indica* rice TY and JXN. Compared to short growth duration *indica* rice TY, planting long growth duration rice JXN and YY could maintain or increase the head rice yields, improve the grain milling and eating qualities, and reduce the adverse impacts on the appearance quality under climate warming. Our findings provide suggestions for the selection of late-season rice cultivars to compensate the warming-induced reductions in grain yields and head rice yields, and to improve rice qualities in future climate warming conditions.

## Data availability statement

The original contributions presented in the study are included in the article/**Supplementary Files**. Further inquiries can be directed to the corresponding authors.

## Author contributions

TY performed most of the experiments and wrote the main manuscript. YaZ and SC wrote the part of the manuscript and revised the manuscript. XT, YoZ, and JZ revised and gave some advice for the manuscript. SH and XP edited the language and modified the main manuscript. All authors contributed to the article and approved the submitted version.
