# Supplementary material for: Grain yield and quality performances of different late-season rice cultivars in response to experimental warming in subtropical China
Source: Front Plant Sci. 2023 May 15;14:1136564. doi: 10.3389/fpls.2023.1136564 (PMC10225640; doi:10.3389/fpls.2023.1136564)
Supplement: Supplementary file 1 [file DataSheet_1.docx]

# Supplementary Materials


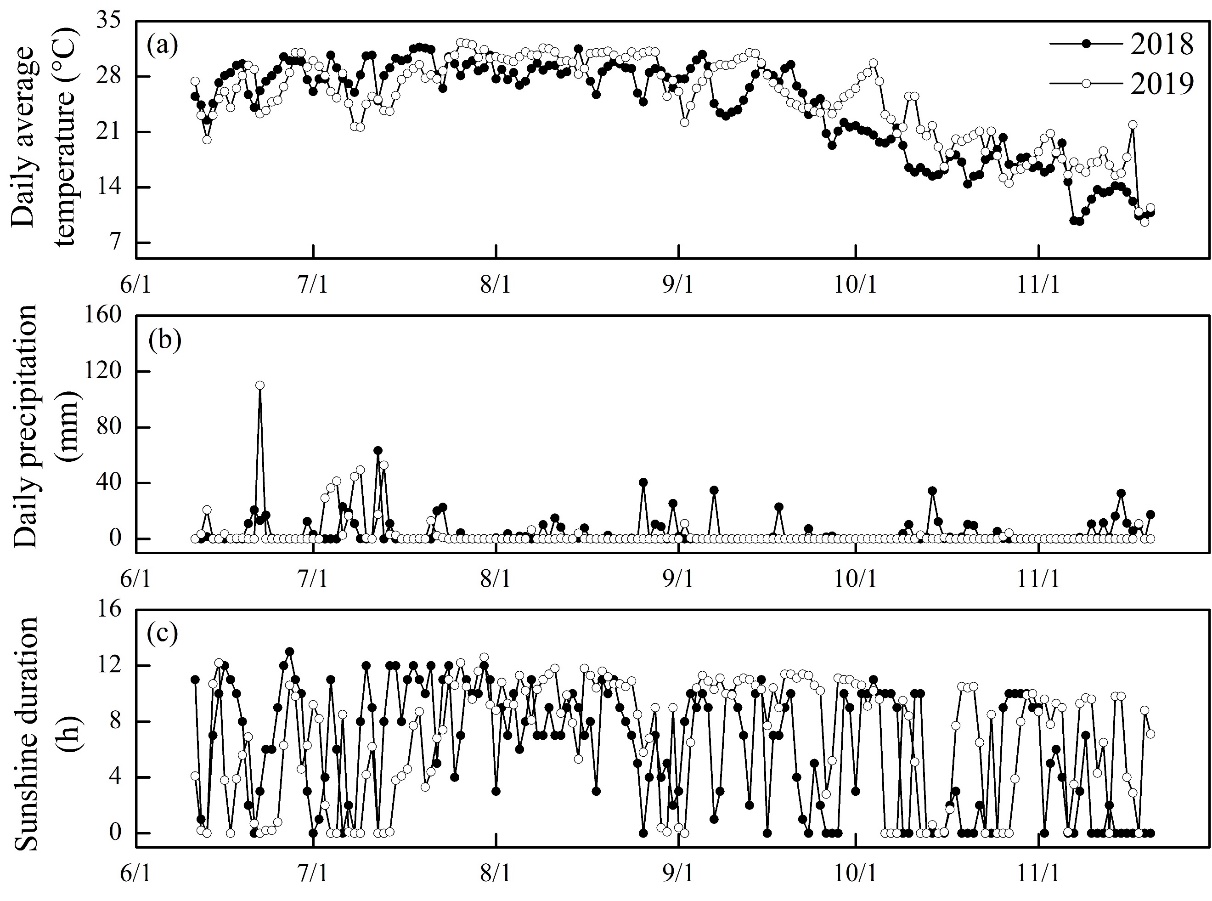


**Figure S1** Daily average temperature (A), daily precipitation (B), and sunshine duration (C) during rice growth period in 2018 and 2019.


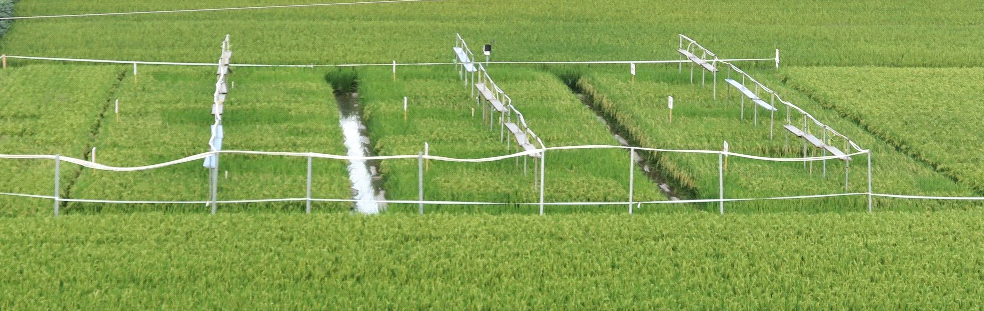

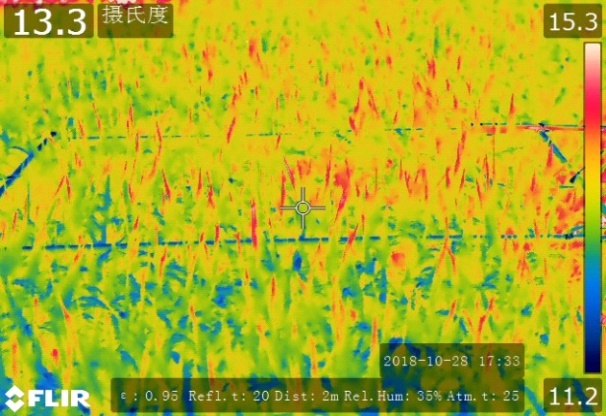

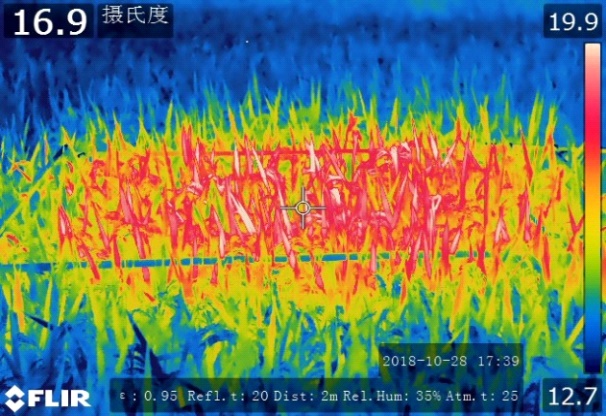


A

B

C

**Figure S2** Free-air temperature increase (FATI) facilities in a double rice cropping system (A) and thermal images of rice canopy (B, ambient treatment; C, warming treatment).


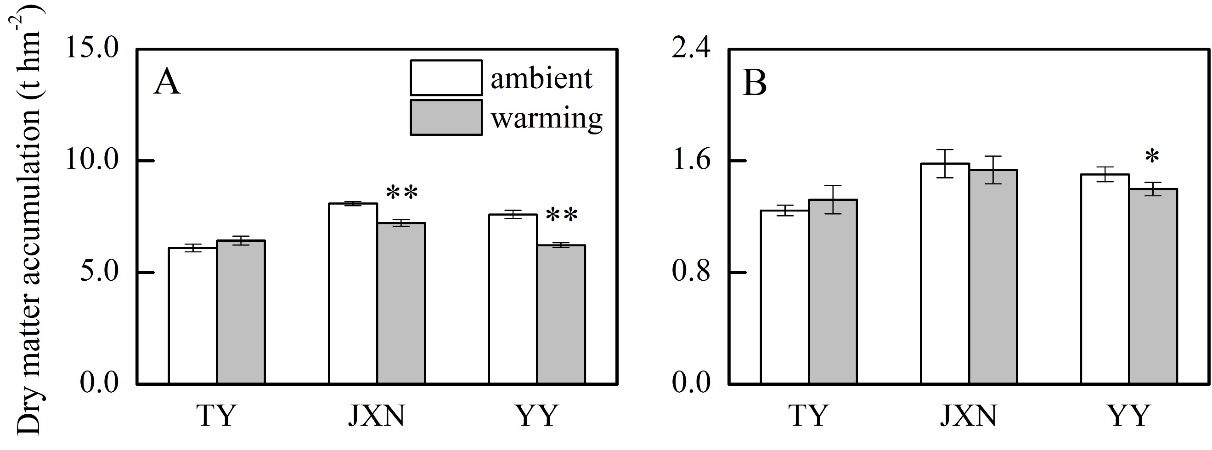
**Figure S3** Rice straw (A) and panicle (B) dry matter accumulation at heading stage as affected by warming in 2019. TY, JXN, and YY indicate Taiyou398, Jiuxiangnian, and Yongyou1538, respectively. Asterisks indicate significant differences between temperature treatments within the same cultivar at 0.001 < *P* ≤ 0.01 (**) or 0.01 < *P* ≤ 0.05 (*).

**Table S1** Sowing, transplanting, heading and maturity date in 2018 and 2019.

| Cultivar | Year | Temperature | Sowing | Transplanting | Heading | Maturity |
| --- | --- | --- | --- | --- | --- | --- |
| TY | 2018 | ambient | 6/21 | 7/16 | 8/28 | 10/10 |
|  |  | warming | 6/21 | 7/16 | 8/31 | 10/12 |
|  | 2019 | ambient | 6/20 | 7/15 | 8/30 | 10/6 |
|  |  | warming | 6/20 | 7/15 | 9/2 | 10/8 |
| JXN | 2018 | ambient | 6/21 | 7/16 | 9/11 | 11/3 |
|  |  | warming | 6/21 | 7/16 | 9/13 | 11/3 |
|  | 2019 | ambient | 6/20 | 7/15 | 9/13 | 11/2 |
|  |  | warming | 6/20 | 7/15 | 9/15 | 11/2 |
| YY | 2018 | ambient | 6/21 | 7/16 | 9/3 | 10/31 |
|  |  | warming | 6/21 | 7/16 | 9/5 | 10/31 |
|  | 2019 | ambient | 6/20 | 7/15 | 9/4 | 11/1 |
|  |  | warming | 6/20 | 7/15 | 9/5 | 11/1 |

TY, JXN, and YY indicate Taiyou398, Jiuxiangnian, and Yongyou1538, respectively.

**Table S2** Average temperature (°C) in the canopy at specific rice growth stages in 2018 and 2019.

| Cultivar | Year | Temperature | Transplanting to panicle initiation | Panicle initiation to initial heading | Initial heading to full heading | Full heading  to maturity |
| --- | --- | --- | --- | --- | --- | --- |
| TY | 2018 | ambient | 32.1±0.1 | 30.2±0.1 | 28.8±0.2 | 25.1±0.2 |
|  |  | warming | 33.3±0.1 | 32.0±0.4 | 31.5±0.4 | 26.6±0.4 |
|  | 2019 | ambient | 31.9±0.2 | 31.3±0.1 | 26.8±0.2 | 26.4±0.2 |
|  |  | warming | 33.3±0.4 | 32.7±0.3 | 28.8±0.2 | 28.6±0.1 |
| JXN | 2018 | ambient | 31.7±0.1 | 29.0±0.2 | 26.4±0.3 | 20.5±0.4 |
|  |  | warming | 33.3±0.2 | 30.4±0.2 | 29.8±0.2 | 22.3±0.2 |
|  | 2019 | ambient | 31.6±0.1 | 29.5±0.1 | 29.3±0.2 | 21.4±0.4 |
|  |  | warming | 33.2±0.3 | 31.5±0.3 | 29.7±0.3 | 23.8±0.3 |
| YY | 2018 | ambient | 31.8±0.3 | 29.8±0.4 | 29.4±0.2 | 21.5±0.1 |
|  |  | warming | 33.4±0.2 | 31.6±0.2 | 29.9±0.1 | 23.5±0.4 |
|  | 2019 | ambient | 31.8±0.2 | 30.5±0.3 | 27.6±0.4 | 22.7±0.3 |
|  |  | warming | 33.4±0.1 | 31.9±0.3 | 31.1±0.3 | 25.1±0.3 |

TY, JXN, and YY indicate Taiyou398, Jiuxiangnian, and Yongyou1538, respectively. Mean ± standard deviation (*n*=3).
